# Human African trypanosomiasis cases diagnosed in non-endemic countries (2011–2020)

**DOI:** 10.1371/journal.pntd.0010885

**Published:** 2022-11-07

**Authors:** Jose R. Franco, Giuliano Cecchi, Gerardo Priotto, Massimo Paone, Augustin Kadima Ebeja, Pere P. Simarro, Abdoulaye Diarra, Dieudonné Sankara, Weining Zhao, Daniel Argaw Dagne

**Affiliations:** 1 World Health Organization, Control of Neglected Tropical Diseases, Prevention, Treatment and Care, Geneva, Switzerland; 2 Food and Agriculture Organization of the United Nations, Animal Production and Health Division, Rome, Italy; 3 World Health Organization, Regional Office for Africa, Brazzaville, Congo; 4 World Health Organization Consultant; Erasmus University Medical Center and Harbour Hospital Rotterdam, NETHERLANDS

## Abstract

**Background:**

Sleeping sickness, or human African trypanosomiasis (HAT), is transmitted by tsetse flies in endemic foci in sub-Saharan Africa. Because of international travel and population movements, cases are also occasionally diagnosed in non-endemic countries.

**Methodology/Principal findings:**

Antitrypanosomal medicines to treat the disease are available gratis through the World Health Organization (WHO) thanks to a public-private partnership, and exclusive distribution of the majority of them enables WHO to gather information on all exported cases. Data collected by WHO are complemented by case reports and scientific publications. During 2011–2020, 49 cases of HAT were diagnosed in 16 non-endemic countries across five continents: 35 cases were caused by *Trypanosoma brucei rhodesiense*, mainly in tourists visiting wildlife areas in eastern and southern Africa, and 14 cases were due to *T*. *b*. *gambiense*, mainly in African migrants originating from or visiting endemic areas in western and central Africa.

**Conclusions/Significance:**

HAT diagnosis in non-endemic countries is rare and can be challenging, but alertness and surveillance must be maintained to contribute to WHO’s elimination goals. Early detection is particularly important as it considerably improves the prognosis.

## Author summary

Human African trypanosomiasis, also known as sleeping sickness, is a parasitic disease transmitted by tsetse flies. The infection can be contracted in several endemic areas in sub-Saharan Africa and, unless correctly diagnosed and properly treated, the disease is usually fatal.

While the vast majority of cases of sleeping sickness are detected in endemic countries, a few are diagnosed in non-endemic ones, notably in travellers or migrants who have visited or resided in the transmission areas. An accurate and early diagnosis of these exported cases is crucial to improve their prognosis. In this study we reviewed cases of trypanosomiasis detected in non-endemic countries in 2011–2020. The data were gathered by the World Health Organization (WHO) thanks to its exclusive distribution of antitrypanosomal medicines.

A total of 49 exported cases of sleeping sickness were detected in the 10 years we studied. Half of them were diagnosed in Europe, 22% in South Africa—a non-endemic country, 14% in North America and 12% in Asia. Only one case was detected in South America. Despite its rarity, travel medicine must maintain alertness on this disease, especially in patients with a history of exposure in endemic areas, with febrile and neuro-psychiatric syndromes and without a clear alternate diagnosis.

## Background

Human African trypanosomiasis (HAT) or sleeping sickness is a neglected tropical disease (NTD) transmitted by tsetse flies (Genus: *Glossina*) and is considered to be endemic in 36 countries of sub-Saharan Africa [1]. Travellers from non-disease endemic countries (non-DEC) who visit areas of HAT transmission are at risk of being infected. At the same time, people living in endemic areas and already infected can travel to non-DEC. Both groups can be diagnosed with the disease. Cases diagnosed in non-DEC are sometimes defined as “exported cases”.

Areas in which HAT can be transmitted are well-known and accurately mapped in disease endemic countries (DEC) thanks to information provided by the national sleeping sickness control programmes (NSSCP). This information is regularly compiled in the Atlas of HAT [2,3], an initiative of the World Health Organization (WHO) in collaboration with the Food and Agriculture Organization of the United Nations (FAO) in the framework of the Programme Against African Trypanosomosis (PAAT). HAT cases can be caused by two different subspecies of pathogen: *Trypanosoma brucei gambiense*, characterized by a more chronic course and transmitted in western and central Africa, and *T*. *b*. *rhodesiense*, which presents a more acute clinical evolution and occurs in eastern and southern Africa [4].

Both forms of the disease are considered lethal, if untreated [5], although long-term latent infections [6,7] and individuals clearing their infections without treatment have been described for gambiense HAT [8]. Given the severity and high case fatality of the disease, all detected cases should receive adequate treatment as early as possible after diagnosis. Manufacturers of anti-HAT medicines (i.e. Sanofi and Bayer) have signed agreements with WHO and are committed to producing all the medicines needed to treat HAT cases. The medicines are donated exclusively to WHO for distribution free of charge to DEC and–when needed–non-DEC in which exported cases are diagnosed. The donation agreement between pharmaceutical companies and WHO was first signed in 2001 and has been extended since then, to ensure the availability of HAT medicines until 2025. Through this arrangement anti-HAT medicines are freely available to all who need them, and WHO is the sole distributor. These medicines are not commercially available, with the exception of pentamidine, which is also produced and distributed for the treatment and prevention of other diseases including *Pneumocystis jiroveci* pneumonia in high-risk patients and leishmaniasis.

Anti-HAT medicines are provided to NSSCPs in DEC on the basis of regular forecasts jointly made with WHO, and according to previously reported cases and planned activities. Distribution is supported by *Médecins Sans Frontières Logistique* (Bordeaux, France), which provides storage, assemblage of treatment kits, packing and shipment services.

In non-DEC, pharmacy services in health facilities that have diagnosed HAT address their requests to WHO for the quantities of medicines needed to treat cases. WHO keeps a stock of medicines at its headquarters in Geneva, which ensures their rapid delivery. In addition, to enable prompt initiation of treatment, which can be critical in some cases of rhodesiense HAT, a few health facilities in non-DEC are supplied with small stocks of antitrypanosomal medicines to act as prepositioned repositories (Table 1).

A review of the data on HAT cases diagnosed and treated in non-DEC during 2000–2010 was published in 2012 [9]. The present paper is a follow-up and focuses on the HAT cases diagnosed in non-endemic countries in 2011–2020.

**Table 1:** Institutions keeping prepositioned stocks of antitrypanosomal medicines for the treatment of HAT cases detected in non-DEC.

Country	Location	Institution	Address
Belgium	Antwerp	Universitair Ziekenhuis Antwerpen (UZA)	UZA Drie Eikenstraat 655 2650 Edegem Belgium
Germany	Wurzburg	Department of Tropical Medicine, Missioklinik	Department of Tropical Medicine, Missioklinik Klinikum Würzburg Mitte gGmbH Salvatorstr. 7 D—97074 Würzburg Germany
	Dusseldorf	Universitätsklinikum, Zentralapotheke	Universitätsklinikum Düsseldorf Zentralapotheke, Arzneimittelausgabe (Geb. 18.23 01.32) Moorenstr. 5 40225 Düsseldorf Germany
Norway	Oslo	Medisinsk klinikk, Oslo universitetssykehus	Sykehusapoteket Oslo, Ullevål Kirkeveien 166 0450 Oslo Norway
Spain	Barcelona	Hospital Clinic	Servei de Farmacia Hospital Clinic Barcelona C/ Villarroel, 170 08036 Barcelona Spain
Switzerland	Basel	FMH Innere Medizin und Tropen- und Reisemedizin, Schweizerisches Tropen- und Public Health Institut (STPHI)	Swiss Tropical and Public Health Institute attn. Ambulatorium Socinstrasse 57 4051 Basel Switzerland
United Kingdom of Great Britain and Northern Ireland	Liverpool	Liverpool University Hospital / NHS Foundation Trust	Royal Liverpool Hospital Prescot Street Liverpool L7 88XP United Kingdom
	London	University College London Hospital / NHS Foundation Trust	Pharmacy Department Mortimer Market Centre off Capper Street London WC1E 6JB United Kingdom
United States of America (USA)	Atlanta	Centers for Disease Control and Prevention	Parasitic Diseases Branch Center for Global Health Centers for Disease Control and Prevention 1600 Clifton Road, NE Atlanta, GA 30329 USA
China	Shanghai	National Institute of Parasitic Diseases, China CDC	Department of Vector transmission Tropical Disease National Institute of Parasitic Diseases, China CDC 207 Rui Jin Er Road Shanghai 200025 China
Japan	Tokyo	National Center for Global Health and Medicine	Disease Control and Prevention Center International Health Care Center National Center for Global health and Medicine 1-21-1 Toyama, Shinjuku-ku Tokyo 162–8655 Japan
South Africa	Johannesburg	Netcare Milpark Hospital	Netcare Milpark Hospital 9 Guild Road Parktown West Johannesburg 2193 South Africa

## Methods

The fact that anti-HAT medicines are not commercially available but obtainable solely from WHO through request allows the Organization to collect epidemiological data on almost all HAT cases diagnosed in non-DEC.

Upon requesting the medicines, health institutions in non-DEC commit to providing WHO with basic epidemiological and clinical data about the patient. Institutions keeping prepositioned repositories are also required to inform WHO of any use of these medicines. The information provided for each HAT case includes: (i) areas of the DEC that the patient visited or where he/she was living; (ii) areas of perceived contacts with tsetse flies; (iii) presumed geographical location of the infection in the opinion of the patient; (iv) laboratory findings, including parasitological and biological tests; (v) main clinical signs and symptoms observed; (vi) treatment administered, adverse events and outcome; and (vii) contact details of the hospital and medical officer in charge of treatment. This information is fully anonymized and does not include any identification of the patient. Such close communication also allows WHO to provide technical advice on HAT case management at the request of the responsible medical officer. The most likely place of infection is inferred from the areas visited and the time spent in them, the reported contacts with tsetse flies, the opinion of the patient about the presumed geographical location of the infection, the onset of clinical signs and symptoms and the existing epidemiological knowledge.

The information received from the non-DEC is shared with the NSSCP of the country where the patient is presumed to have been infected, with a view to reinforcing control and surveillance activities in transmission areas. The cases reported in non-DEC are also included by the NSSCP in their national reports and statistics, and they are taken into account when the DEC is evaluating the possible elimination of the disease.

The reporting arrangement described herein is linked to the exclusive distribution of anti-HAT medicines by WHO, which since 2001 has allowed an exhaustive database of HAT cases diagnosed and treated in non-DEC to be compiled. The information is also integrated in the WHO Atlas of HAT and these cases are included in the statistics of the country where the patient was infected. The present paper provides updated information for 2011–2020. Information provided to WHO by health facilities in non-DEC was complemented through a literature review.

## Results

**Fig 1:**
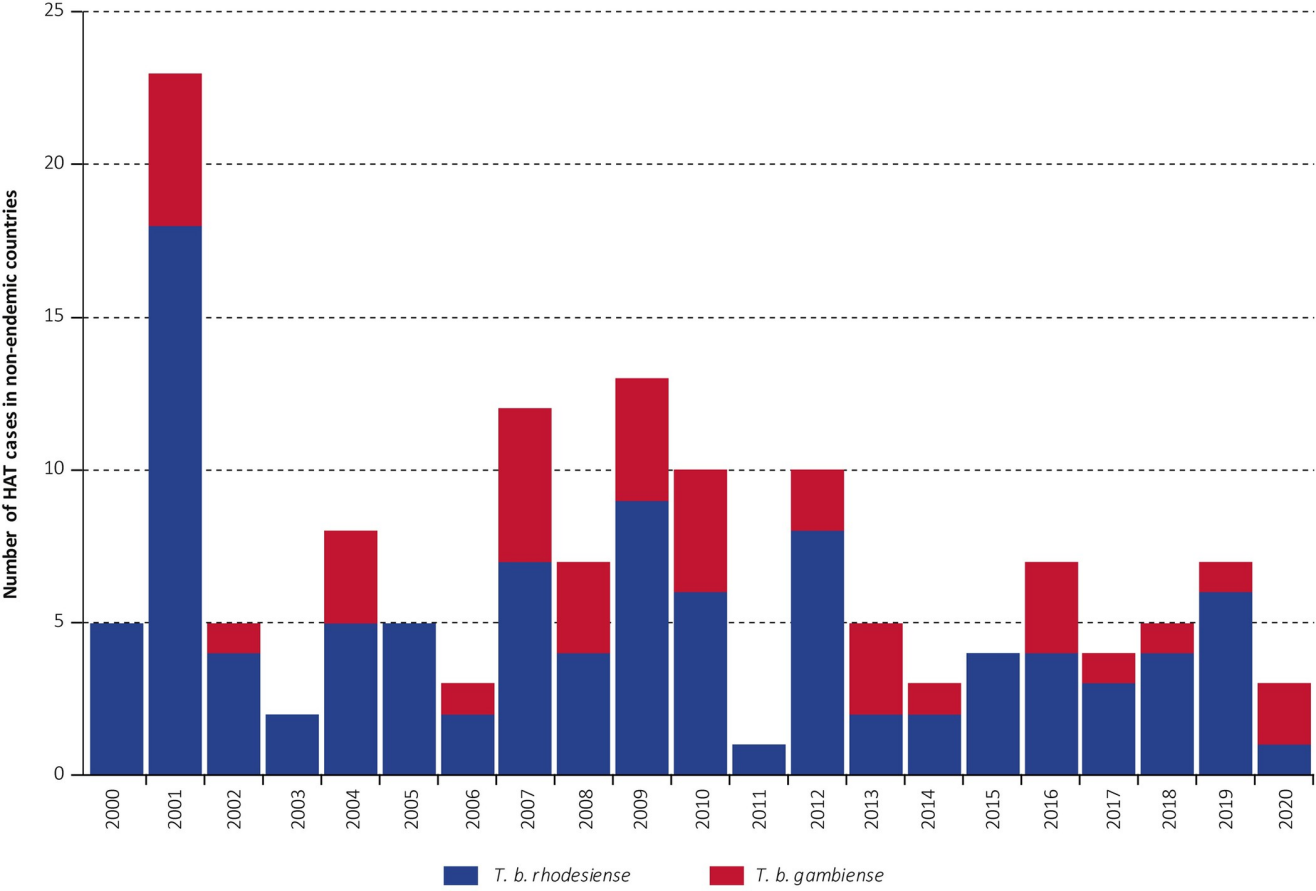
Cases of HAT detected in non-DEC. 2000–2020.

During 2011–2020, 49 HAT cases were diagnosed and reported in 16 non-DEC (mean of 4.9 cases/year): 71% (35/49) were caused by the rhodesiense form of the disease (Table 2) and 29% (14/49) were due to the gambiense form (Table 3). These figures correspond to a 44% reduction from 2001–2010 during which period a mean of 8.8 cases/year was detected in non-DEC (Fig 1). In the same period, the total reduction of HAT cases diagnosed worldwide was 79% (from 155 961 cases to 33 096 cases) [3].

**Table 2:** Cases of rhodesiense HAT diagnosed in non-DEC. 2011–2020.

Year Month	Place of diagnosis	Place of infection	Sex Age	Activity	Diagnosis	Stage	Chancre	Treatment	Reference
2011									
Feb	London United Kingdom	Mana Pools NP Zimbabwe	M	Tourist	Blood smear	1		Suramin	
2012									
Jan	Frankfurt Germany	Masai Mara NR Kenya	M 61	Tourist	Blood smear	1	Yes	Suramin	[25,42,43]
Feb	Antwerp Belgium	Masai Mara NR Kenya	M	Tourist	Blood smear	1	Yes	Suramin	[44,45]
Jul	Buenos Aires Argentina	North Luangwa NP Zambia	M 65	Hunter*	Blood smear	1	Yes	Pentamidine / Suramin	[59]
Oct	Minneapolis, MN USA	Lake Kariba Zimbabwe	F 64	Tourist	Blood smear	1		Pentamidine	[60]
Oct	Houston, TX USA	North Luangwa NP Zambia	M 57	Hunter	Blood smear	1	No	Suramin	
Dec	Eskilstuna Sweden	Ngorongoro CA United Republic of Tanzania	F 55	Tourist	Blood smear	1	No	Pentamidine / Suramin	[61]
Dec	Johannesburg South Africa	Kazumbe (Petauke) Zambia	M 37	Hunter	Blood smear	1	Yes	Suramin	[62]
Dec	Chambery France	Kasanka NP Zambia	M 22	Pilot (tourist company)	Blood smear	1	No	Suramin	[63]
2013									
Jan	Johannesburg South Africa	Kasanka NP Zambia	M 42	Conservation worker	Blood smear	1	No	Suramin	[64]
Jan	Johannesburg South Africa	Mkomazi NP United Republic of Tanzania	M 42	Conservation worker	Blood smear	1	No	Suramin	
2014									
Nov	London United Kingdom	South Luangwa NP Zambia	M 53	Tour operator	Blood smear	1	No	Suramin	
Nov	Pretoria South Africa	Murchison Falls NP Uganda	M 52	Missionary	Blood smear	1	Yes	Suramin	[64]
2015									
Jan	Mumbai India	Kafue NP Zambia	M 46	Local tourist*	CSF	2	No	Melarsoprol	[65]
Jul	Ottawa Canada	Lower Zambezi NP Zambia	F 59	Tourist	Blood smear	1	No	Pentamidine / Suramin	[66]
Sept	Barcelona Spain	Serengeti NP United Republic of Tanzania	F 49	Tourist	Blood smear	1	Yes	Pentamidine / Suramin	[67,68]
Oct	Antwerp Belgium	Queen Elizabeth NP Uganda	F 53	Tourist	Blood smear	1	Yes	Suramin	[69,70]
2016									
Jan	Ancona Italy	Serengeti NP United Republic of Tanzania	M 37	Tourist	Blood smear	1	No	Pentamidine / Suramin	
Apr	Bergen Norway	Murchison Fall NP Uganda	M 64	Humanitarian worker	Blood smear	1	No	Pentamidine / Suramin	
Oct	Leiden, The Netherlands	Serengeti NP United Republic of Tanzania	F 56	Tourist	Blood smear	1	No	Pentamidine / Suramin	[71]
Dec	Baltimore (MD) USA	South Luangwa NP Zambia	M 48	Tourist	Blood smear	1	No	Pentamidine / Suramin	[72,73]
2017									
May	Amsterdam The Netherlands	Serengeti NP United Republic of Tanzania	M 58	Tourist	Blood smear	1	Yes	Suramin	[74,75]
Aug	Frankfurt Germany	South Luangwa NP Zambia	F 50	Tourist	QBC	1	Yes	Suramin	[76]
Aug	Fujan China	Serengeti NP United Republic of Tanzania	F 45	Tourist	Blood smear	1	Yes	Pentamidine / Suramin	[77]
2018									
Jan	Johannesburg South Africa	South Luangwa NP Zambia	M 52	Tourist	Blood smear	1	Yes	Suramin	[78–80]
Jan	New Delhi India	Murchison Falls NP Uganda	F 28	Tourist	Blood smear	1	Yes	Suramin	[81]
Nov	Rotterdam The Netherlands	Vwaza Marsh WR Malawi	F 66	Tourist	Blood smear	1	Yes	Suramin	[45,46]
Dec	Johannesburg South Africa	Vwaza Marsh WR Malawi	M 24	Naturalist volunteering in NP*	Blood smear	2	Yes	Suramin	[47]
2019									
Mar	Johannesburg South Africa	Munyamadzi GR / West Petauke GMA Zambia	M	Hunter	Blood smear	1	Yes	Suramin	[82]
Jun	Johannesburg South Africa	Nkhotakota WR Malawi	M 36	Fishing	Blood smear	1	No	Suramin	[83,84]
Sep	Johannesburg South Africa	Murchison Falls NP Uganda	M 23	Tourist	Blood smear	1	Yes	Suramin	[85]
Oct	Johannesburg South Africa	Vwaza Marsh WR Malawi	F 41	Conservation worker	Blood smear	1	Yes	Suramin	[48]
Oct	Johannesburg South Africa	Vwaza Marsh WR Malawi	M	Naturalist	Blood smear	1	Yes	Suramin	[49]
Dec	Goteborg Sweden	West Petauke GMA Zambia	M 64	Hunter	Bone marrow	2	No	Melarsoprol	
2020									
Jan	Ahmedabad India	Murchison Falls NP Uganda	F 35	Tourist	Blood smear	1	No	Suramin	[86]

GR; game reserve; GMA: game management area; NP: national park; NR: nature reserve; WR: wildlife reserve.

* Died during treatment

**Table 3:** Cases of gambiense HAT diagnosed in non-DEC. 2011–2020.

Year Month	Place of diagnosis	Place of infection	Sex Age	Activity	Diagnosis	Stage	Chancre	Treatment	Reference
2012									
	Porto Portugal	Quiçama (Bengo) Angola	M 41	Construction worker	PCR	2	No	Melarsoprol	
Sep	Montreal Canada	Kinshasa Democratic Republic of the Congo	F 64	Immigrant visiting her country of origin	CTC	1	No	Pentamidine	[87]
2013									
Jan	Paris France	Libreville Gabon	M 29	Trader	PCR / IFAT / CATT	2	Yes	NECT	[88]
Jun	Tours France	Kinshasa Democratic Republic of the Congo	F 22	Immigrant	CSF	2	No	Eflornithine	[89,90]
Jun	Tours France	Kinshasa Democratic Republic of the Congo	M 1	Immigrant	CSF	2	No	Eflornithine	[90]
2014									
Sep	Nanjing China	Port Gentil Gabon	M 45	Timber worker	Blood smear	2	No	Eflornithine	[11,91–93]
2016									
Aug	Limoges France	Bandundu Democratic Republic of the Congo	F 21	Immigrant	CSF	2	No	NECT	[94,95]
Sep	London United Kingdom	Warri (Delta State) Nigeria	F 58	Immigrant (missionary)	PCR / IFAT	2	No	NECT	[53]
Dec	Meaux France	Dubréka Guinea	M 55	Immigrant	PCR	2	No	NECT	[96,97]
2017									
Aug	Shanghai China	Libreville Gabon	M 59	Rural worker	Blood smear	2	Yes	NECT	[55,98]
2018									
May	Creteil France	Dubréka Guinea	F 45	Immigrant	Blood smear	2	No	NECT	[99]
2019									
Sept	Dallas (TX) USA	Mamfé Cameroon	M 51	Immigrant (missionary)	Bone marrow	2	No	NECT	[100]
2020									
Jun	Berlin Germany	Dubréka Guinea	M 26	Immigrant (musician)	CTC	2	No	Eflornithine	
Sep	Baltimore (MD) USA	Mamfé Cameroon	F 49	Immigrant	PCR	2	No	NECT	[54]

CATT: card agglutination test for trypanosomiasis; CSF: cerebrospinal fluid; CTC: Capillary tube centrifugation; IFAT: immunofluorescent antibody test; NECT: nifurtimox-eflornithine combination therapy; PCR: polymerase chain reaction.

### Non-endemic countries of diagnosis

**Fig 2:**
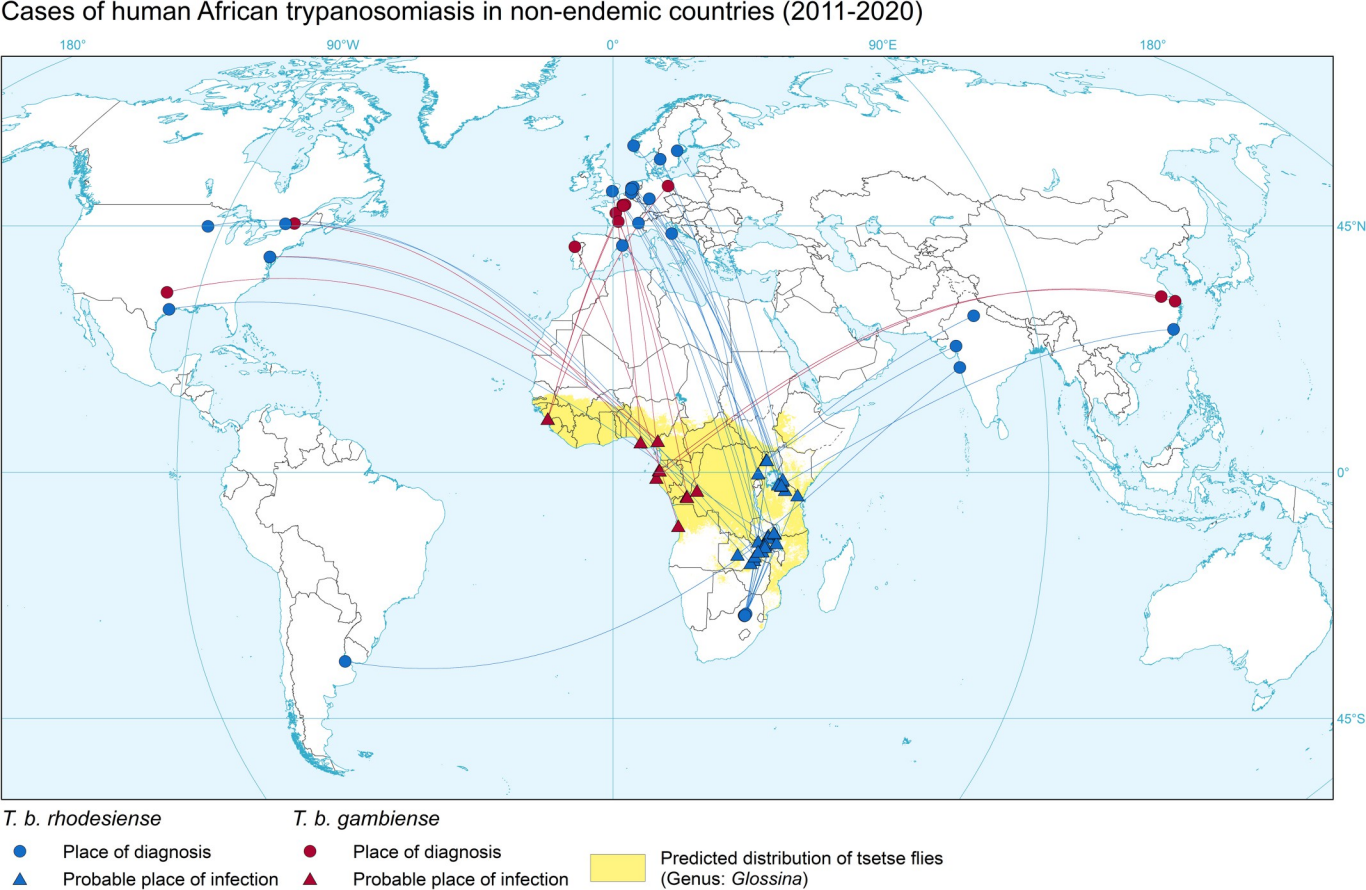
Cases of HAT diagnosed in non-DEC. 2011–2020. Circles represent the place of diagnosis; triangles indicate the probable place of infection. Gambiense HAT (red), rhodesiense HAT (blue). The base layers used in this map are the FAO Global Administrative Unit Layers (GAUL) https://data.apps.fao.org/map/catalog/srv/eng/catalog.search#/metadata/9c35ba10-5649-41c8-bdfc-eb78e9e65654 and FAO Inland water bodies in Africa https://data.apps.fao.org/map/catalog/srv/eng/catalog.search;jsessionid=B7AF7A215B16770A1A67C65D97FF21CA?node=srv#/metadata/bd8def30-88fd-11da-a88f-000d939bc5d8.

Some 49% (24/49) of the HAT cases detected in non-DEC were diagnosed in Europe, 22% (11/49) in one African non-DEC (South Africa), 14% (7/49) in North America, 12% (6/49) in Asia and one case in South America (Fig 2).

As in 2000–2010, South Africa was the non-DEC diagnosing the highest number of cases, all of them being rhodesiense HAT. This results from the country’s proximity to endemic areas and the frequency of medical evacuations to South Africa from other sub-Saharan African countries. Since 2004 the National Institute for Communicable Diseases in Johannesburg has served as a centre for HAT surveillance, while also monitoring the supply and distribution of medicines; it also provides technical advice on case management, not only within South Africa but also to other countries [10].

After South Africa, the other non-DEC reporting the highest numbers of HAT cases were France (14%, 7/49, six of which were gambiense HAT) and the United States of America (10%, 5/49, three of which were rhodesiense HAT). Other European countries accounted for another 35% (17/49) of cases: Germany (3), the Netherlands (3), United Kingdom of Great Britain and Norther Ireland (3), Belgium (2), Sweden (2), Italy (1), Norway (1), Portugal (1) and Spain (1). The remaining cases were diagnosed in China (3), India (3), Canada (2) and Argentina (1). Of note is that there was no previous record of HAT diagnosed in China [11]. The three cases included in the present study were all Chinese nationals, two of them workers infected with *T*. *b*. *gambiense* and one a tourist infected with *T*. *b*. *rhodesiense*. These cases can be linked to the recent growth in investment in and exchanges between China and African countries that has resulted in increases in imported NTDs in China [12–14]. Recommendations have been made to ensure adequate treatment of future possible cases of HAT and other NTDs in China [15].

### Endemic countries of infection

**Fig 3:**
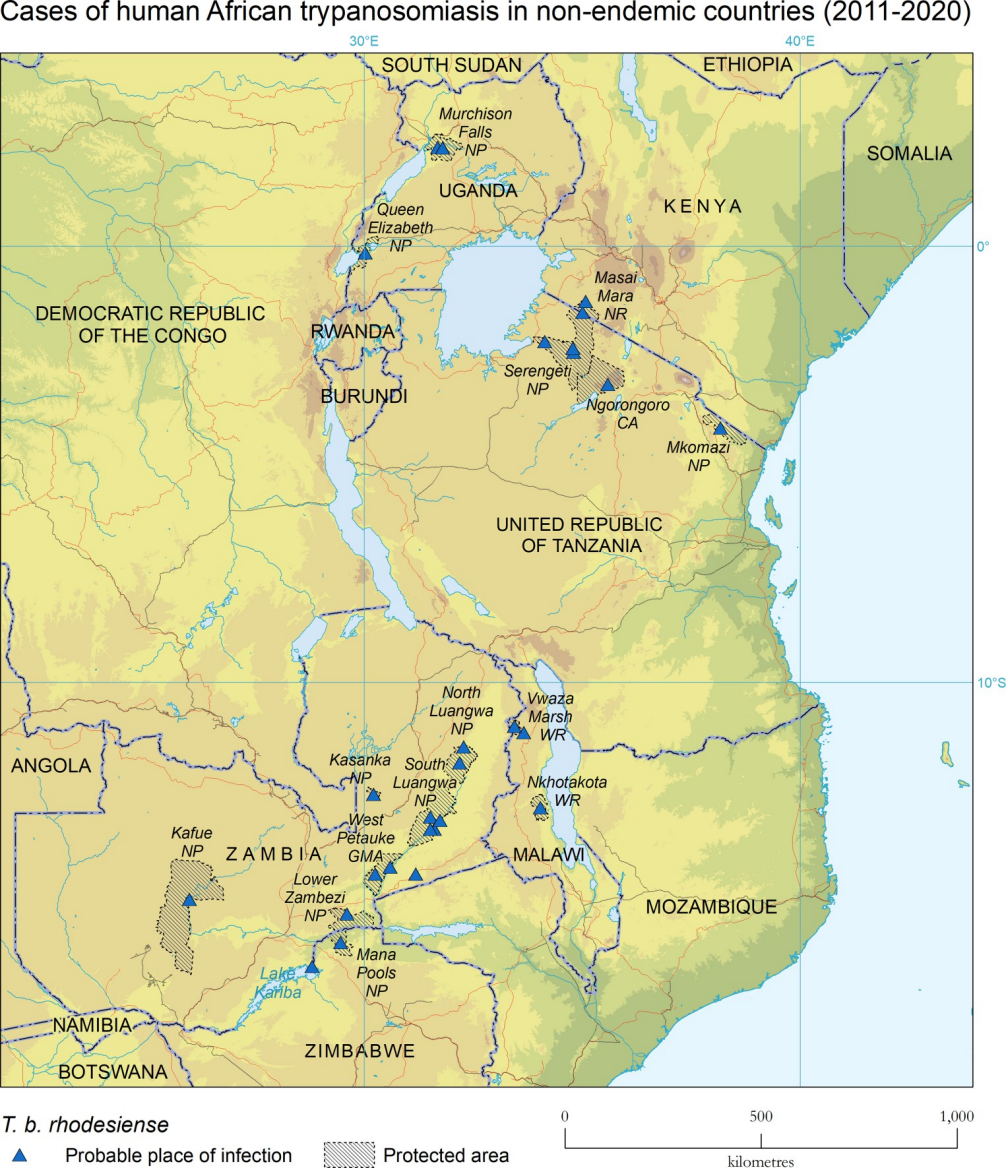
Probable place of infection of cases of rhodesiense HAT diagnosed in non-DEC. 2011–2020. The base layers used in this map are the FAO Global Administrative Unit Layers (GAUL) https://data.apps.fao.org/map/catalog/srv/eng/catalog.search#/metadata/9c35ba10-5649-41c8-bdfc-eb78e9e65654, Shuttle Radar Topography Mission (SRTM) https://www.usgs.gov/centers/eros/data-tools, FAO Inland water bodies in Africa https://data.apps.fao.org/map/catalog/srv/eng/catalog.search;jsessionid=B7AF7A215B16770A1A67C65D97FF21CA?node=srv#/metadata/bd8def30-88fd-11da-a88f-000d939bc5d8, and FAO Rivers of Africa https://data.apps.fao.org/map/catalog/srv/eng/catalog.search;jsessionid=B7AF7A215B16770A1A67C65D97FF21CA?node=srv#/metadata/b891ca64-4cd4-4efd-a7ca-b386e98d52e8.

For rhodesiense HAT cases diagnosed in non-DEC during 2011‒2020 (Table 2), the country of infection accounting for most cases was Zambia, with 37% (13/35) or 19% of the total number of HAT cases reported in the country during the same period (i.e. 13/69 [3]). The United Republic of Tanzania accounted for 20% (7/35, or 33% of the 21 cases reported in the country in the same period), Uganda 17% (6/35, representing 2% of the 330 cases reported in the country in the same period) and Malawi 14% (5/35, representing 1.3% of the 375 cases reported in the country in the same period). Other infections occurred in Kenya (2 cases, which represent the only cases reported in Kenya during the period) and Zimbabwe (2 cases, representing 8% of the 24 cases reported in the country in the same period). The cases were infected in protected areas such as national parks (NP), wildlife reserves (WR), national reserves (NR), conservation areas (CA), game reserves (GR) and game management areas (GMA) (Fig 3). Some of these protected areas accounted for several cases, such as South Luangwa NP (4 cases), North Luangwa NP (2), Kasanka NP (2) and West Petauke GMA (2) in Zambia, Serengeti NP in the United Republic of Tanzania (5), Murchison Falls NP in Uganda (5), Vwaza WR in Malawi (4) and Masai Mara NR in Kenya (2). The cases in Malawi were linked in time and space to a general outbreak of the disease [3].

For gambiense HAT, exported cases reported during the study period were infected in the Democratic Republic of the Congo (4), Gabon (3), Guinea (3), Cameroon (2), Angola (1) and Nigeria (1).

### Diagnosis

Rhodesiense HAT was diagnosed in non-DEC by demonstrating the presence of parasites. In 32 cases (91%), trypanosomes were found in blood smears, in three cases they were detected through examination of cerebrospinal fluid (CSF), and in two instances through bone marrow and buffy coat examination (one each). Trypanosomal chancre was present in around half of the rhodesiense HAT cases (18); information on the possible presence of chancre was not available in two cases.

The time lag between exposure and diagnosis was well described in 21 cases of rhodesiense HAT (60%). In seven cases it was around one week, in 10 cases it was around 2 weeks, in three cases it was around 3 weeks and in one case it was more than 1 month.

Gambiense HAT cases were diagnosed by combining parasitological, serological and molecular investigations. Parasites were found through parasitological tests in nine cases (64%); in particular, the parasites were observed in blood smear examination (3 cases), cerebrospinal fluid (3), capillary tube centrifugation of blood (2) and bone marrow aspirate (1). In five cases diagnosis relied on polymerase chain reaction (PCR) [16] in combination with serological tests [i.e. immunofluorescent antibody test (IFAT) [17], card agglutination trypanosomiasis test (CATT) [18], rapid diagnostic test (RDT) [19–20], immunotrypanolysis (TL) [21] and enzyme-linked immunosorbent assay (ELISA) [22,23]]. The WHO collaborating centre for HAT diagnosis at the Institute of Tropical Medicine Antwerp (Belgium) plays a key role in supporting HAT diagnosis in non-DEC by performing some of these more specific tests in referred samples. The probable time of infection was clearly established in 10 of the gambiense HAT cases (71%): in four cases it was less than 1 year before diagnosis (between 3 and 10 months), in three cases it was around 1 year, in two cases diagnosis occurred about 2 years after exposure and in one case more than 4 years after exposure. Chancre was described in only two of the 14 gambiense HAT cases.

Regarding the stage of the disease, 91% (32/35) of rhodesiense HAT cases were diagnosed in first stage and 9% (3/35) were diagnosed in second stage. For gambiense HAT, 7% of cases (1/14) were diagnosed in first stage and 93% (13/14) in second stage.

### Activity

All 35 rhodesiense HAT cases diagnosed in non-DEC were linked to exposure to the wildlife reservoir in protected areas: 20 cases were in tourists visiting these areas for short periods, five of whom were hunters, one a fisherman and seven were working in the protected areas for long periods (five as conservationists or researchers, one as an aircraft pilot for tourism and one as a tour operator); the remaining two were missionary or humanitarian workers who occasionally travelled to protected areas for rest and recreation.

Of the 14 gambiense HAT cases diagnosed, 10 were people originally from DEC currently living in non-DEC, of whom seven had settled in non-DEC several years earlier and who visited their country of origin for short periods (e.g. holidays, family visits, business); two had recently established in non-DEC. One case was a 1-year-old child who had never been in any DEC but whose mother originated from one and was diagnosed with HAT at the same time; in this case vertical transmission is assumed.

Four of the gambiense HAT cases were nationals from non-DEC who had been working in DEC for extended periods, one for business (a trader), one as a river sailor in a timber enterprise, one in agriculture and another in construction.

### Treatment and outcome

Of the 14 cases of gambiense HAT, only one that was detected in first stage was treated with pentamidine. Cases diagnosed in second stage were treated with either nifurtimox-eflornithine combination therapy (8 cases), eflornithine monotherapy (4) or melarsoprol (1). No death was reported among these cases.

Of the 35 cases of rhodesiense HAT, 32 were diagnosed in first stage of which 31 were treated with suramin and one with pentamidine. In nine cases treated with suramin, treatment was initiated with pentamidine while the supply of suramin was being dispatched. This approach is warranted because of the fast progression and acute presentation of the disease, as it allows parasitaemia to be rapidly reduced [24–25]. Two of the three rhodesiense HAT cases diagnosed in second stage were treated with melarsoprol; the third received initial treatment with suramin to clear parasitaemia and died before treatment with melarsoprol could be started. Three cases of rhodesiense HAT died during treatment, showing a case-fatality rate of 8.6%: one fatality could be ascribed to a terminal stage of the disease linked to a late diagnosis following misdiagnosis in different health facilities, a second to the toxicity of melarsoprol (encephalitic reaction) and the third to severe acute disease complications.

### Cases published in scientific journals

Of the 49 HAT cases diagnosed in non-DEC during 2011–2020, 19 (39%) were published in scientific journals. These publications usually focus on clinical aspects and travel medicine issues and contribute also to raising awareness of this unusual diagnosis. Scientific publications were more frequent for gambiense HAT (9/14) than for rhodesiense HAT cases (10/35). Of the 35 rhodesiense HAT cases diagnosed, 22 (63%) were reported via epidemiological networks including the Communicable Diseases Communiqué of the National Health Laboratory Services, South Africa (http://www.nicd.ac.za), ProMed (http://www.promedmail.org), GeoSentinel (http://www.geosentinel.org) and Eurosurveillance (https://www.eurosurveillance.org/). No case of gambiense HAT was reported in these networks.

In 2012, a gambiense HAT case was diagnosed in the United Kingdom [7] in a patient originally from Sierra Leone. It is assumed that this individual had been infected at least 29 years earlier, because their last visit to a DEC was in 1983. Given the time lag between detection and probable infection for this case of at least 29 years, the case was neither included in the statistics of HAT occurrence kept by WHO since 1990 [3,26] nor in Table 2. Of note is that the last autochthonous case from Sierra Leone was reported in 1982 [27–28].

During 2011–2020, 11 additional papers were published (5 in 2012, 1 in 2013, 2 in 2014 and 3 in 2016) concerning cases diagnosed during 2000–2010; therefore they were not considered for this paper [28–39].

## Discussion

Agreements between WHO and the producers of antitrypanocidal medicines ensure access to adequate treatment for all cases of HAT diagnosed in both DEC [40] and non-DEC. WHO ensures availability, distribution and use of these medicines. As an important added value, the exclusive distribution system allows systematic collection of valuable epidemiological information. This information is integrated with the bulk of the data collected on monitoring of transmission patterns, which informs targeted surveillance in DEC. In some instances, information from non-DEC can highlight “grey areas” where reports of autochthonous cases are rare or absent (e.g. Murchison Falls NP in Uganda, South Luangwa Valley NP and Kafue NP in Zambia). This provides important information on the risk of transmission in these areas and on the possible exposure of local populations, triggering reinforced control and surveillance measures. On a few occasions, cases in non-DEC reflect the occurrence of epidemic outbreaks of rhodesiense HAT in particular areas (e.g. Vwaza Marsh NP in 2019–2020 [25,41–44], Masai Mara NR in 2012 [45–49] and Serengeti WR in earlier periods [50]).

The present review confirmed that, owing to international travel and the movement of human populations, HAT can be detected across the globe. This is despite the fact that disease transmission has dramatically abated in endemic areas over the past two decades [3]. Epidemiological information on exported cases of HAT can help to maintain awareness about this differential diagnosis in travellers. It is also valuable as a sentinel within the global HAT surveillance system.

Treatment was ensured for all cases of HAT in non-DEC that were reported to WHO. As opposed to the general pattern in DEC, the majority of exported cases in non-DEC are due to *T*. *b*. *rhodesiense* from exposure to the wildlife reservoir in protected areas that are frequented by foreigners for leisure or professional activities. Transmission generally occurs from infected wild animals to humans through the tsetse fly and, given the high density of flies in many of these areas, even brief exposure can carry a risk. Conversely, transmission of *T*. *b*. *gambiense* occurs in remote rural areas that foreigners rarely visit and do not usually reside in for extended periods.

During 2011–2020, a total of 33 096 HAT cases were reported globally, of which 32 275 (97.5%) were gambiense HAT and 821 (2.5%) were rhodesiense HAT [3]. Cases detected in non-DEC represent only 0.15% of the number of cases reported globally, but they constitute 4% of all rhodesiense HAT cases.

As rhodesiense HAT is an acute disease with high parasitaemia that can be clearly linked to travel to DEC, cases are diagnosed quickly and relatively easily by blood smear a few weeks after infection. Diagnosis of gambiense HAT is more complex because of typically low parasitaemia and non-specific, chronic symptoms which resemble many other pathologies. Recently, however, new molecular methods have simplified the diagnosis of gambiense HAT in sophisticated laboratories. Nevertheless, molecular diagnosis remains laborious and time-consuming, requires expertise and is often done only when cases have already reached the second stage. Epidemiological elements of clinical anamnesis (e.g. geographical tracking of the patient’s travel history and wanderings, exposure to tsetse fly bites), together with adequate laboratory tests not only to look for parasites but also to check antibodies and the presence of parasite DNA or RNA, can play a key role in the differential diagnosis of HAT [10,51,52]. Early diagnosis and availability of appropriate treatment are the main elements to ensure a full recovery.

Interestingly, HAT diagnosis in countries with more sophisticated technology that is not always available in DEC generates useful information on the clinical and pathogenicity aspects of the disease. These more advanced tools include imaging techniques (e.g. Magnetic Resonance Imaging (MRI) [53] and computed tomography (CT) scan [54,55]) and the analysis of biochemical parameters.

Overall, as previously documented [56], a contrast is observed in exported HAT cases: rhodesiense HAT usually affects more affluent populations of non-DEC, whereas gambiense HAT is normally detected in African migrants with limited economic means. In some cases, these people are in precarious situations and may not be covered effectively by the health system in non-DEC, which can contribute to a delayed diagnosis.

This paper focuses on cases that fit the classical definition of HAT, and it does not include sporadic cases of atypical trypanosomiasis in humans [57,58]. Atypical cases can be due to species of trypanosomes that are different from those usually affecting human beings, the latter being *T*. *b*. *gambiense* and *T*. *b*. *rhodesiense*, as well as *T*. *cruzi* as the cause of Chagas disease.

## Conclusions

Despite the rarity of sleeping sickness in non-DEC, it is important that travel medicine services in non-DEC maintain awareness of HAT risk among travellers and migrants presenting with a history of exposure in endemic areas, with febrile and neuro-psychiatric syndromes and without a clear alternate diagnosis. Descriptions of tsetse bites or even the presence of cutaneous lesions (chancre) can aid diagnosis, mainly for rhodesiense HAT. Early detection is particularly important as it considerably improves the prognosis.

The availability of HAT medicines through WHO guarantees timely access to treatment in non-DEC and should therefore be sustained. This exclusive distribution arrangement also ensures notification of cases to WHO, thus providing valuable epidemiological information for action in DEC and contributing to ongoing efforts to eliminate the disease.
